# Self-Medication With Antibiotics: An Element Increasing Resistance

**DOI:** 10.7759/cureus.30844

**Published:** 2022-10-29

**Authors:** Chetna Sachdev, Ashish Anjankar, Jayesh Agrawal

**Affiliations:** 1 Community Medicine, Jawaharlal Nehru Medical College, Datta Meghe Institute of Medical Sciences, Wardha, IND; 2 Biochemistry, Jawaharlal Nehru Medical College, Datta Meghe Institute of Medical Sciences, Wardha, IND

**Keywords:** low- and middle-income country, prevention of antimicrobial resistance, inappropriate use of antibiotics, self medication, antimicrobial resistance

## Abstract

Self-medication refers to the consumption of drugs such as antibiotics by individuals based on their own experience and knowledge, without consulting a doctor either for diagnosis or prescription. The inappropriate use of antibiotics is the primary source of antibiotic resistance (AR) development in microorganisms. As a result, some specific types of microorganisms that are naturally resistant to antibiotics have become considerably more common. Self-medication poses a danger to the advantages of antibiotics since it results in financial burdens on low and middle-income countries (LMICs), management failures, the evolution of antibiotic-resistant bacterial serotypes, and a higher risk of contamination of the general population by such tensions. Antibiotic misuse puts patients at risk for adverse drug reactions, false symptom relief, and the rise of drug-resistant microorganisms. It carries many health risks, chiefly in LMICs. These risks are linked to various factors, including a shortage of medical experts, low-level healthcare facilities, unregulated medication delivery, and negative public perceptions of doctors. The primary issue with self-medication is that majority of the population is uninformed of the harmful consequences of antibiotic resistance and how they might donate to it by self-diagnosing and self-treating with antibiotics. Antibiotic self-medication remains a common practice in society, and educational attainment significantly affects the frequency of this behavior. The article aims to educate the people by showing the development and plausible future to decrease antibiotic misuse. It also tells about the various challenges and prevention of this preceding problem.

## Introduction and background

Most people believe bacteria to be microorganisms or pathogens causing trauma to their already ageing viscera. Antibiotics counter these bacteria and are the silver bullet solution to many medical problems. Without prescriptions, people also unknowingly consume antibiotics to counter "viral" diseases like cough, cold, and flu. 

Antibiotics are medicines against bacteria that kill or inhibit their growth and multiplication. They can be administrated through various routes of administration (i.e., oral, topical, or injections) [1]. They can be considered a non-renewable resource for the body since their effect reduces on their successive use. Patients who do not finish the entire course of antibiotics run a higher chance of developing resistance. Patients frequently stop receiving the treatment as they start to feel better. 

Self-medication is diagnosing and prescribing drugs by an ordinary person without taking a physician's advice leading to the unnecessary expense of time and money, morbidities due to the adverse effects, and eventually, the development of antibiotic resistance (AR) by a reduction in the efficacy of antibiotics. It is a widespread practice all over the globe, especially in developing nations, and is seen as a substitute for those who are unable to afford the costs of healthcare amenities. The primary causes of self-medication include a shortage of medical supplies, long travel times to medical facilities, unfriendly attitudes of healthcare workers toward patients, and prior knowledge of the illness and its treatments. All these factors prevail in low and middle-income countries and add to the development of resistance [2]. Antibiotic resistance is a global issue that poses a therapeutic conundrum for doctors from all specialties, like drug selection for a particular illness and dosage selection. The absence of antibiotic alternatives, ignorance, and avoidance of the hazardous effects of misuse, and lack of knowledge on the part of the general population have led to the emergence of this global crisis [3].

## Review

Antibiotics

The advent of antibiotics created an optimistic attitude toward fighting infectious diseases. The antibiotics work by showing various inhibition processes like inhibition of protein biosynthesis, inhibition of folic acid metabolism, and inhibition of DNA metabolism; some may also target bacterial cell wall synthesis [4]. Development of antibiotic resistance by the bacteria is by different mechanisms such as the prevention of gathering antibiotics either by reducing its intake or increasing the excretion from tissues, i.e., changing the outer membrane permeability, antibiotic inactivation, target modification, and bypassing the metabolic path. A better interpretation of the antibiotic resistance mechanism can help combat the smartness of the bacteria [5].

Keeping in mind the patient's pharmacokinetics and the dynamics of the drugs choosing and prescribing the most appropriate medication is the key to optimally treating the infections along with reduced adverse effects and fewer chances of developing antibiotic resistance [6]. Individuals' adherence to prescriptions and careful use of antibiotics are among the most prevalent factors that can reduce the development of drug resistance; thus, there is a demand to change individuals' behaviors regarding the use of antibiotics [7,8]. 

Antibiotic resistance

One of the many causes of the emergence of antibiotic resistance is an interruption in the ongoing medical treatment of a disease. This could be because people tend to forget their dosage and drug consumption when they start to feel better after their symptoms subside. The bacteria have become more resistant due to this inadequate treatment in terms of dose and duration [9]. The regular and incorrect administration of antibiotics for self-limiting diseases, such as the common cold, flu-like symptoms, diarrhea, and sore throat, is recognized as the most significant contributor to antimicrobial resistance growth [10]. The resistance has increased organisms' virulence and led to the widespread prevalence of diseases caused by bacteria. 

Broad-spectrum antibiotics are widely and unnecessarily utilized in healthcare settings due to the absence of early identification of pathogenic bacteria and their antimicrobial sensitivity patterns in patients with bacteremia and other severe diseases [11]. Antibiotic resistance has many adverse effects, including its influence on the selection and dosage of antibiotic regimens, classes of antibacterial drugs that may be used, and the use of less effective treatments. The increased frequency with which empirical antibiotic selections for the treatment of common diseases are modified is a direct result of the evolution in antibiotic resistance that has taken place over the last few decades [12].

The public health sector faces a global challenge in developing strategies to prevent and fight the emergence of antibiotic resistance. Globalization and growth in worldwide travel and trade both unavoidably produce opportunities for the quick transmission of hazardous diseases and make the issue of antibiotic resistance considerably more challenging to solve [13]. Even the last-resort antibiotics are no longer effective in treating infections caused by resistant microorganisms.

Epidemiology of antibiotic resistance

World Health Organization has estimated that 10 million deaths can occur by 2050, which could be attributed to an increase in AR [14]. In addition to mortality and morbidity, it can result in higher resource usage and costs, altered guidelines, and decreased hospital activities. The unseemly interaction between drug dispensers and clients leads to inappropriate antibiotic dispensing. Antibiotics are seen more in countries where infectious diseases are prevalent, like low and middle-income countries (LMICs).

In high-middle-income countries like China, Brazil, South Africa, and Libya, antibiotic resistance is a public health crisis due to the evolution of modern medicine. Antibiotic resistance poses many threats, like the failure of the therapy, an increase in infectious diseases, population biology alterations, and bacterial evolution [15]. Indian research found that among medical experts, self-medication is more frequently done to treat mild ailments [16]. In contrast, analysis from Malaysia and Pakistan indicated that one of the primary drivers of self-medication was knowledge of the available treatments [17].

A study initiated amongst health experts showed that antibiotics are the second most common type of medicine used by self-medication, the first being analgesics [18]. Self-medication is particularly common in underprivileged communities [19]. It is a simple and necessary medical option in many LMICs where healthcare facilities fall short of international standards and are even quite expensive. The ease with which prescribed medications can be obtained as over-the-counter (OTC) medications from any pharmacy also help to encourage self-medication in low and middle-income countries. Additionally, a proliferation of over-the-counter medicines present for the management of common diseases results from lax medical regulations [3]. The development and dissemination of organisms resistant to several drugs seem to have picked up a significant amount of speed during the last fifty years, according to an analysis of organisms and epidemiological data. Antibacterial agents were discovered around this period, and their usage became more familiar at about the same time [20]. According to the data in the table, the most commonly used antibiotic is amoxicillin [21]. The population extensively uses amoxicillin for various sorts of bacterial infections such as upper respiratory tract infections (caused by Streptococcus pyogenes, Streptococcus pneumonia, and Hemophilus influenzae), meningitis, and urinary tract infections since they have a broad spectrum (Table 1).

**Table 1:** The percentage use of self-medicated antibiotics of various antibiotics SMA - self-medicated antibiotics Source: [21]

Name of antibiotic	Percentage use for SMA
Amoxicillin	40.9
Ampicillin + Cloxacillin	16.2
Doxycycline	4.8
Metronidazole	17.2
Cotrimoxazole	6.1

Reasons for antibiotic misuse

Studies found that people chose to self-medicate with antibiotics for the following reasons: extra cost suffered from facility custodies; presumed information on antibiotics use extensive waiting stretch required to refer to health care facilities; experience with similar signs or antibiotics; disease supposed as minor by the patient; instruction from friends or relatives; absence of time to refer; monetary limitation; impolite behavior of health workers; lack of self-assurance in the effectiveness of antibiotics; lack of confidence in the efficacy of antibiotics; experience with similar symptoms and a layman often cannot even acknowledge if the given medicine is antibiotic or not, contributing to confusions and negligence in completing the antibiotic therapy [22].

**Figure 1:**
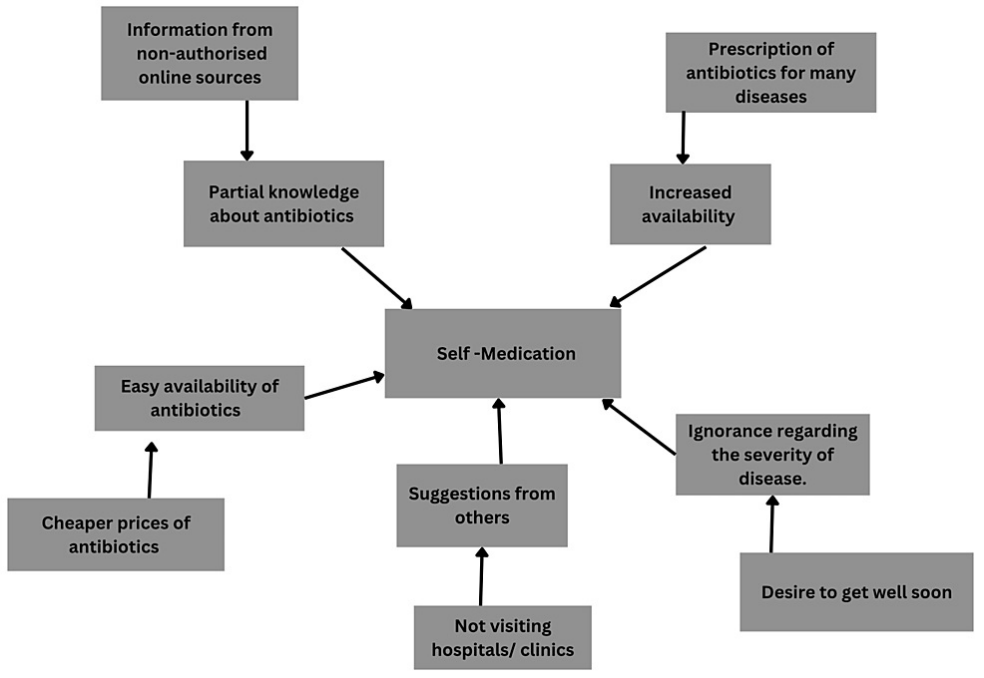
Reasons for self-medication Source: [25]

The selling of antibiotics and medications on Schedule H without a valid prescription is prohibited by the Indian Drugs and Cosmetics Act. However, numerous studies in the nation have indicated a significant incidence of antibiotics being sold over the counter, leading to self-medicated behavior [23,24]. Adding to all this, patients consider themselves capable of achieving and maintaining their own health; even after getting diagnosed with a chronic disease, they rarely seek advice from a physician. A vast portion of the population uses medications like histamine H2-receptor blockers, antifungals, oral contraceptives, and topical corticosteroids without seeking medical advice, along with frequently using antibiotics. This pattern reveals an absence of knowledge regarding administering antibiotics and other medications to avoid the troublesomeness of going to the doctor [25]. Some more reasons for the frequent practice of antibiotic self-medication are easy accessibility of antibiotics, unawareness about the severity of the illness, and easily accessible in terms of time and money, information about the antibiotics, and ideas from other people (Figure 1) [26].

Along with self-medication, the other factors leading to antibiotic resistance are the unseemly interaction between drug dispensers and clients that leads to inappropriate dispensing of antibiotics, poor public health, the unauthorized buying of antibiotics, high occurrence of infectious diseases, less access to appropriate antibiotics, low-quality diagnostic tools and prophylactic treatment also encourages the use of antibiotics [27]. Further, an insufficient understanding of the significance of finishing antimicrobial rehabilitation and awareness of antimicrobial resistance has been described in several studies. It has been linked to the general public's indiscriminate use of antibiotics [28].

Prevalence of self-medication

Self-medication may help treat acute illnesses that do not need consultation from a doctor, or recurring disorders diagnosed previously during earlier consultations. When done responsibly, it may facilitate faster relief to the patient by providing prompt medication access. It can be a cost-effective option in settings of limited sources, particularly in LMICs. However, inappropriate self-medication subjects the patient to several risks and drawbacks like adverse drug reactions, development of antibiotic resistance, masking of a more severe underlying condition and failure to consult a doctor in time [29]. It is still practiced all over the globe. The prevalence of self-medication in different regions is shown in Table 2 [30-36].

**Table 2:** Prevalence of self-medication

First author	Year	Title of the article	Study region	Sample size	The reported prevalence of self-medication %
Elden [30]	2020	Risk factors of antibiotics self-medication practices among university students in Cairo, Egypt	Egypt	600	77.7
Amin [29]	2019	Prevalence of antimicrobial self-medication among patients attending two hospitals in the Buea Health District, Cameroon	Cameroon	329	68.4
Badger-Emeka [31]	2018	Evaluation of the extent and reasons for increased non-prescription antibiotics use in a university town, Nsukka Nigeria	Nigeria	400	86
Banerjee [32]	2016	Self-medication practice among preclinical university students in a medical school from the city of Pokhara, Nepal	Nepal	488	81.4
Jimenez-Nunez [33]	2016	Impact of a training action on the prevalence of self-medication among students from the Faculty of Education Sciences at the University of Malaga	Spain	249	72.7
Williams [34]	2016	Self-medication practices among undergraduate nursing and midwifery students in Australia: a cross-sectional study	Australia	120	91.7
Gelayee [35]	2017	Self-medication pattern among Social Science University students in Northwest Ethiopia	Ethiopia	385	32.7
Jakaria [36]	2017	Evaluation of self medication among students from different universities in Chittagong, Bangladesh	Bangladesh	439	52.2

Consequences of antibiotic misuse 

Self-medication results in antibiotic misuse, which has many consequences like adverse drug reactions, drug interactions, increased morbidity and mortality, and the emergence of AR [37]. It decreases the chance of making a proper diagnosis, allows for the contraction of the disease, and delays the initiation of appropriate therapy [38]. It can also lead to numerous health risks, particularly in LMICs. These risks are connected to various factors, including poverty, inaccessibility to hospital settings, a scarcity of medical professionals, inadequate quality of healthcare facilities, non-regulated delivery of medicines, and patients' misconception about practitioners [39].

People who do not obtain appropriate treatment on time are at an increased risk for a more severe course of the disease or a fatal outcome. Additionally, these patients remain communicable for extended periods, increasing the probability of transmission of resistant microorganisms if infection prevention measures are not executed [40]. In a study, about one-third of patients who were administered antibiotics did not adequately obey their antibiotic medication, and one-fourth of patients retained surplus drugs for forthcoming usage, which shows a broad outline of poor antibiotic administration and prescription behaviors [41].

Prevention of antibiotic misuse

Methods of prevention include providing health education which will help to draw attention to the issues caused by antibiotic self-medication, especially among graduates and professionals. These issues involve using a doctor's previous prescription to obtain antibiotics, treating minor illnesses like a sore throat with antibiotics, switching antibiotics during self-medication, and stopping antibiotics when symptoms go away. In order to educate and spread awareness, specific campaigns to inform the public about various conditions and the value of seeking medical care from hospitals can be organized by the state and central governments.

Specific other ways by which populations can overcome self-medication practices include availing medical supplies and personnel in public facilities-also and constructing more medical facilities close to communities to cut down on travel time to access health care. Healthcare professionals must modify how they view patients [42]. They should also inform their patients and care providers about the emotional factors, such as improved inspiration, patient instruction, health goals, and increased social care, that can help them improve medication adherence [3]. Public health practitioners could also create specific interventions that would help to lessen the burden of antibiotics self-medication and improve measures for controlling antibiotics sales if they know the sources of antibiotics self-medication [43].

The issue of unused antibiotics can be resolved in the following ways: monitor and regulate doctors' prescribing practices to prevent overprescribing; instruct patients to administer antibiotics exactly as given through effective health education programs; forbid the sale of antibiotics without a prescription in pharmacies, and encourage patients to discard unused antibiotics [44]. Avoiding antibiotics that promote the spread of resistance genes, selecting resistant pathogen variants from susceptible pathogens, and eliminating antibiotic-susceptible normal flora are all important ways to reduce antibiotic resistance [45].

Examining and considering patients' modified consultation behavior and other behavioral components should be one way to aim in future campaigns. These could include delayed prescription of antibiotics. Shortening the course of antibiotics prescribed to three to five days will axiomatically reduce the use of leftover antibiotics. Educated people can be given a message of not reusing antibiotics without proper diagnosis and knowing the disease completely [46]. Even if their development is disturbingly sluggish, the creation of novel antibiotic drugs with an expanded range of action can potentially reduce some of the detrimental impacts of antibiotic resistance [47].

Challenges faced in the prevention of antibiotic resistance

It is crucial to inform people regarding the development of antibiotic resistance in microorganisms and the limited management options available for infections related to drug resistance to prevent it from becoming an even bigger global crisis. The emerging problem of antibiotic resistance is not only a worry for the healthcare sector; if it is not contained, it could potentially harm a nation's ability to grow economically [3].

The pharmaceutical industry must also overcome the dearth of new antibiotics being developed to treat bacterial infections. The growing crisis of antibiotic resistance and the threat of bacterial infections becoming fatal once more place the effectiveness of presently accessible antibiotic drugs in grave jeopardy. Lack of economic encouragement for research and strict supervisory requirements are two of the most important reasons why new drugs to treat bacterial infections with high case fatality rates have not been discovered [48].

There are components in the healthcare system on both the supply and demand sides that contribute to the massive levels of antibiotic overuse for self-limiting infections. Due to healthcare providers' supply-side financial dependence on prescription sales, hospitals and community health centers unavoidably overprescribe antibiotics. The pharmaceutical industry is also considered a supply-side actor in the issue of antibiotic misuse. Most antibiotics are used outside hospitals worldwide, and access to antibiotics without a prescription is relatively frequent [49,50]. The number of people looking for antibiotics online may have increased since the beginning of the COVID-19 era, so people avoid going to the doctor in person [51].

## Conclusions

Self-medication is increasing at an alarming rate globally, particularly in LMICs, and is the primary cause of the development of antibiotic resistance in microorganisms. Different factors contribute to the practice of self-medication, like partial knowledge of antibiotics, easy availability of antibiotics, quick relief from acute illnesses, and ignorance regarding the severity of the disease. There is a need to educate the general population about the inappropriate administration of antibiotics, such as using them for minor illnesses like sore throat, interrupting the treatment when symptoms subside, and switching antibiotics without consulting a doctor. The evolution in AR that occurred over time is the primary reason for the increased frequency with which initial regimens of choice for treating common diseases are modified. Thus, the emerging AR crisis is not only a concern for the healthcare industry since it could also affect a country's economic growth if not controlled.
